# Generating reliable hypomorphic phenocopies by RNAi using long dsRNA as diluent

**DOI:** 10.17912/micropub.biology.000269

**Published:** 2020-07-01

**Authors:** Kimberly N Bekas, Bryan T Phillips

**Affiliations:** 1 Interdisciplinary Graduate Program in Genetics, University of Iowa, Iowa City, IA; 2 Department of Biology, University of Iowa, Iowa City IA

**Figure 1:**
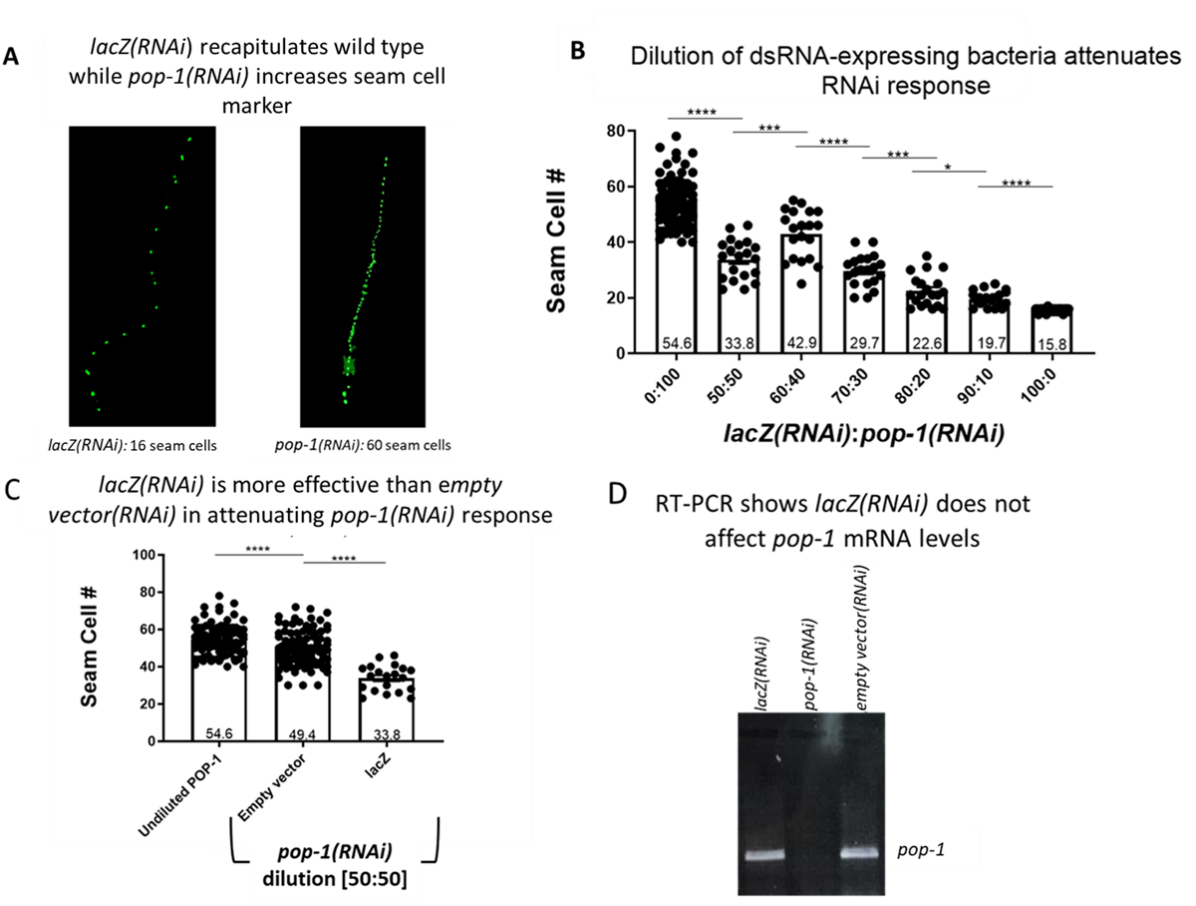
**Longer dsRNA is a more effective dilutor for bacterial mixing RNAi** **(A)** Representative images of *C. elegans* seam cells expressing the *wIs51* (SCMp::GFP) transgene show worms subjected to *lacZ(RNAi)* are identical to worms grown on OP50 bacteria (not shown)while worms subjected to *pop-1(RNAi)* result in an expected increase in seam cell number **(B)** Dilution of *pop‑1(RNAi)* with *lacZ(RNAi)* gives a range of additional seam cells with a 50:50 dilution showing the intermediate response (*lacZ:pop-1* RNAi dilutions of 0:100, n=100; 50:50, 60:40, 70:30, 80:20, 90:10, n=20; 100:0, n=50 where n values are the sum of five biological replicates). Means are shown at the bottom of each bar. **(C)** Seam cell output was measured in animals treated with undiluted *pop-1(RNAi)* (n=100), *pop-1(RNAi)* diluted at a 50:50 ratio with bacteria containing an empty pL4440 vector (n=100), or *pop-1(RNAi)* diluted at a 50:50 ratio with bacteria containing a *lacZ* insert (n=20), where n values are the sum of five biological replicates. The latter two samples are similarly diluted, yet a significant difference in seam cell output exists when diluting with *lacZ* compared to empty vector. **(D)** Reverse-transcriptase PCR amplification of *pop-1* mRNA isolated from worms subjected to *lacZ(RNAi)*, *pop-1(RNAi)* or *empty vector(RNAi)* shows no effect of *lacZ(RNAi)* on *pop-1* mRNA levels*.* (*p-value= 0.046; ***p-value = 0.0003-0.008; ****p-value<0.0001 via two-tailed unpaired t-test).

## Description

In *C. elegans*, RNAi is performed by delivering double-stranded RNA (dsRNA) to the worm via feeding (Timmons and Fire 1998), injection (Fire *et al.* 1998) or soaking (Tabara, Grishok, and Mello 1998). In feeding RNAi, sequence of a target gene is placed between two oppositely oriented promoter sequences active in *E. coli* (e.g. T7 in the pL4440 vector (Timmons and Fire 1998)). Subsequent T7 polymerase expression in *E. coli* provides sense and anti-sense transcription of the target gene sequence, generating dsRNA. The bacteria producing the dsRNA is consumed by the worm and the dsRNA distributed to *C. elegans* cells (Timmons and Fire 1998), resulting in efficient target gene knockdown.

When performing RNAi by feeding, one can achieve a range of knockdown phenocopies by diluting the bacteria producing dsRNA targeting the gene of interest with similarly prepared bacteria producing dsRNA from empty pL4440 vector. The ability to dilute the level of knockdown of a target gene is useful when undiluted RNAi causes developmental defects, or if a partial loss of function is preferred over a full gene knockdown. While mixing with empty vector bacteria sufficiently dilutes the target gene bacteria fed to the worm, it may not be sufficient to dilute the silencing RNAs loaded into RNAi riboproteins. Specifically, an “empty” vector plasmid produces a short amount of dsRNA consisting of only 185bp of polylinker sequence. Thus, there will be few dsRNA segments in worm cells after empty vector RNAi feeding. Therefore, dilution of a desired target gene RNAi vector, which often produces closer to 1000bp of dsRNA, with empty vector plasmid may result in a preponderance of RNAi riboproteins loaded with target gene dsRNA, minimizing the dilution effect. Here, we asked if dilution with a plasmid generating a longer dsRNA than empty vector would be more effective at diluting the target gene mRNAs leading to a partial loss of function phenotype.

To begin, we identified a gene that displayed allele-dependent variations in expressivity. We decided to evaluate the effect of *pop-1*/TCF knockdown on seam cell output since knockdown of POP-1-mediated transcriptional repression via feeding RNAi is known to result in an increase from the wild-type number of 16 seam cells to as many as 60 seam cells (Banerjee *et al.* 2010). The seam cells undergo several rounds of Wnt-signaled asymmetric cell divisions where low nuclear POP-1/TCF in the posterior daughter confers seam cell fate while higher POP-1/TCF in the anterior daughter results in hypodermal cell fate (Baldwin and Phillips 2018; Baldwin, Clemons, and Phillips 2016; Lam and Phillips 2017; Lin, Hill, and Priess 1998; Takeshita and Sawa 2005). To utilize seam cell number as an output, we performed our experiments in the strain JR667 which marks the seam cell fate with GFP from the *wIs51* transgene (Figure 1A). To determine if a RNAi vector encoding longer dsRNA sequences was a more effective dilutor than the pL4440 empty vector, we inserted a portion of the *E. coli lacZ* gene, which is absent from the *C. elegans* genome, into pL4440. The resulting *lacZ(RNAi)* vector produces 1255bp of dsRNA (1070bp of *lacZ* plus 185bp polylinker),which is a similar length to our *pop-1(RNAi)* vector that generates 1292bp dsRNA (1107bp of *pop-1* cDNA plus 185bp polylinker).

In order to find an RNAi dose that results in an intermediate *pop-1* phenocopy that can be used as a baseline for different dsRNA dilution protocols, we showed that bacteria mixing of *pop-1(RNAi)* with *lacZ(RNAi)* at different ratios corresponded to different increases in seam cell output (Figure 1B). Generally, highly diluted *pop-1(RNAi)* bacterial cultures corresponded to a mild increase in seam cells (e.g. 19.7 seam cells at the 90:10 dilution of *lacZ(RNAi):pop-1(RNAi)*) whereas less dilute *pop-1(RNAi)* bacteria corresponded to a larger increase in seam cell number (e.g. 33.8 seam cell at a 50:50 dilution of *lacZ(RNAi):pop-1(RNAi)*) (Figure 1B). Since our goal was to obtain an intermediate seam cell phenocopy, we proceeded with our empty vector versus *lacZ* comparison using a 50:50 dilution ratio.

We next asked if an empty vector or a vector containing *lacZ* would affect seam cell output differently when diluted at the same ratio. Indeed, we saw that when *pop-1(RNAi)* bacteria were diluted 50% with *lacZ*, the worms resulted in significantly fewer seam cells (average, 33.8 seam cells) compared to dilution at the same ratio with empty vector (average, 49.4 seam cells) (Figure 1C). This result indicates that bacterially supplied *lacZ* dsRNA was more efficient in attenuating the *pop-1* RNAi response compared to dsRNA produced from empty vector. Although *lacZ* dsRNA should not target the *C. elegans* transcriptome, we wanted to confirm that *lacZ(RNAi)* was not affecting *pop-1* levels. To do this, we performed reverse-transcriptase PCR on worms subjected to RNAi of *lacZ*, *pop-1*, or empty pL4440 vector. As expected, we only saw a change in *pop-1* expression in the *pop-1(RNAi)* condition (Figure 1D).

Together, our data show that bacterial mixing with a vector generating longer dsRNA serves to better dilute knockdown of a target gene compared to empty vector. The differences in effective dilution may be attributed to a similar number of dsRNA segments being produced from both the *lacZ(RNAi)* and *pop-1(RNAi)* vectors. In contrast, the minimal dsRNA produced by the *empty vector(RNAi)* may result in significantly less empty vector loaded RNAi riboproteins compared to target gene loaded riboproteins. In summary, the length of dsRNA generated by the diluting vector should be similar to the length of dsRNA produced by the target gene vector for effective bacterial mixing RNAi dilution.

## Methods

The *lacZ* sequence was PCR amplified, inserted into pL4440, and sequence verified. RNAi constructs were expressed from *E. coli* HT115(DE3) bacteria, which were grown on plates containing 1 mM IPTG to induce T7 polymerase activity at convergent promoters (Timmons and Fire 1998). Simultaneous RNAi was carried out by growing the two different RNAi bacterial strains to the same optical density and mixing volumes in the ratios indicated before plating. RNAi was induced by L1 synchronized feeding (Timmons and Fire 1998) for 75 hours at 15°C and resulting L4 worms were immobilized using 1mM Levamisole on 5% agarose pads for live imaging. Six L4 worms not harvested for imaging were used to isolate RNA (Ly, Reid, and Snell 2015). Specifically, the six cleaned worms were transferred into 6ul of worm lysis buffer, frozen at -80°C then incubated in a thermocycler at 65 °C for 10 minute followed by 85 °C for 1 minute, to inactivate proteinase K. The worm lysate was then used immediately for semi-quantitative reverse-transcriptase PCR using SuperScript® III First-Strand Synthesis System with oligo(dT) followed by 25 rounds of *pop-1* cDNA-specific amplification. In the genome, a large intron is located between primer sites, which distinguishes genomic DNA from cDNA.

## Reagents

Strain JR667 [*unc-119*(*e2498*::*Tc1*) III; *wIs51* (SCMp::GFP + *unc-119*) V]

SuperScript® III First-Strand Synthesis System

Primers

Primer 5’ to 3’ sequence

*lacZ* F: AACGTCGTGACTGGGAAAA

*lacZ* R: GACCTGACCATGCAGAGGAT

*pop-1* mRNA F: ATGATGGCCGACGAAGAGCTCGGCG

*pop-1* mRNA R: CATCGTATGCATCATTGCTGCTTG
